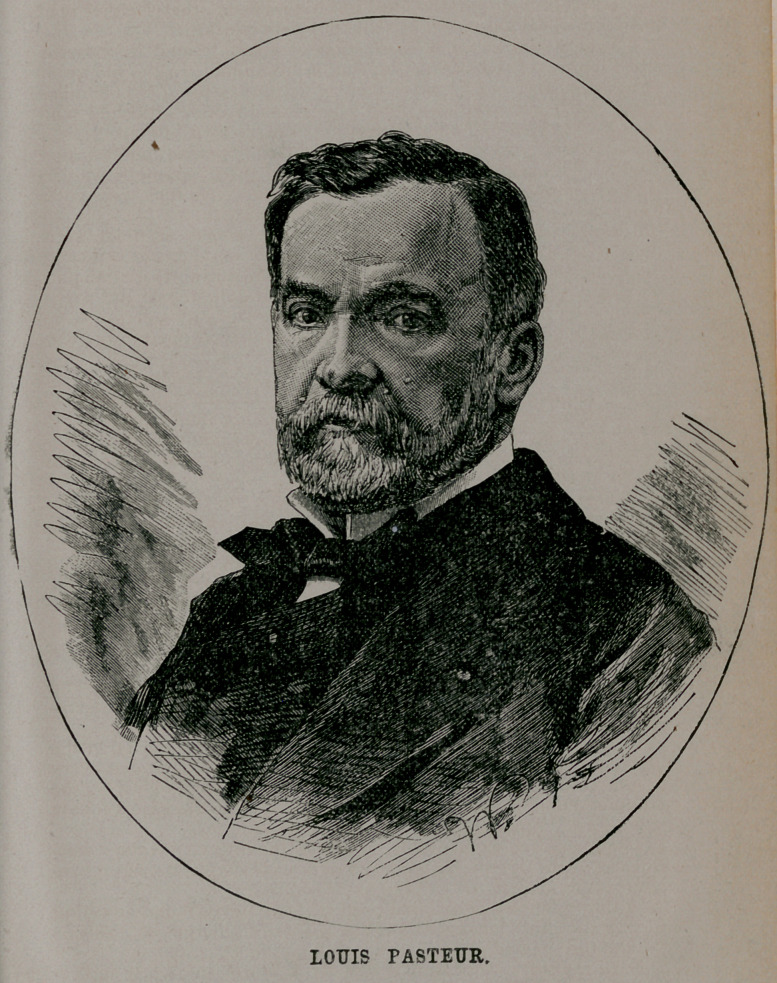# Abstracts and Gleanings

**Published:** 1885-01-20

**Authors:** 


LOUIS PASTEUR.

### ABSTRACTS AND GLEANINGS.

**Louis Pasteur.**—Louis Pasteur was born in Dole in 1822 and was appointed Teacher in Chemistry at Besancon and then at Dijon, and finally was appointed Professor of Chemistry at Strasburgh in 1849. In 1857 he conducted the Normal School in Paris and in 1863 was appointed Professor of Chemistry at the Sorbonne. He was compelled to resign his position, as one side of his body became paralyzed, but he gradually regained his health sufficiently to be able to take up his chemical researches, and in order to enable him to give full attention to his studies the French Government has granted an annual pension of twelve thousand francs since 1874, which was raised to twenty thousand francs recently. In one of his first works he discovered that crystalized organic substances, although having the same chemical properties, have decidedly different physical properties, especially in the relation to the refraction of light. He made many valuable discoveries in relation to fermentation, and was able to prove that the process of fermentation, that is, the conversion of sugar into alcohol and carbonic acid, is due to the vitality of the yeast germ. In this matter the celebrated chemist Liebig was his opponent, but Pasteur's experiments were so numerous and new, and at the same time so absolutely exact, that his success was assured. He finally conceived the idea of making experiments to ascertain whether yeast germs, fermentation, mold, etc., should originate of themselves in fluid. His experiments proved beyond any doubt that this was not possible and thus settled this question of long standing. He also discovered a method of preserving wine and beer by heating it for about thirty minutes to from 46° to 48° centigrade, whereby the yeast germs are destroyed and prevent further decomposition of the liquid. Since 1870 Pasteur had given all his attention to contagious diseases such as anthrax, chicken cholera and rabies of dogs. All the diseases are caused by parasites or microbes, and he claims that by inocculating part of the poison in very small quantities and very much diluted into the system a person is less apt to be affected by these diseases than those who have not been thus inoculated. Toussaint previously made experiments with the blood of animals suffering from anthrax, but Pasteur has succeeded in raising anthrax bacilli in a drop of blood by preserving the germs upon certain substances. The strength as a poison was diminished to such an extent as not to cause any disease. Injections of this diluted poison protected animals to such an extent that very few suffered from anthrax, where formerly entire herds were killed.

The latest experiments Pasteur has made are in relation to the rabies of dogs, and during the first months of this year he notified the Paris Academy that by inoculating dogs with microbe organisms, they have been protected from the bites of rabid dogs. The details of the results of these experiments are so well known that they need no further mention here.—*New England Medical Monthly.*

[For the cut which illustrates this sketch we are indebted to the courtesy of the publishers of the Scientific American.—*Ed. Rec.*]

**Shall We Bleed?**—R. L. Hinton, M. D., Prescott, Ark., says in The Therapeutic Gazette: A few years ago I was called to see J. C., aet. 17, in the town of F., late in the afternoon, a young man of slight, delicate frame, but usually in good health, as he was at the time of this attack. His father met me at the gate, told me that in my absence from town, early in the morning he had called in Dr. M., (will state that I was the family physician) that he had left some medicine which his son had been taking all day, but had been growing worse. I told him to send for Dr. M., and in the meantime I learned that the boy had been complaining some little for a day or two, that in the early part of the previous night he was taken with a chill, which was soon followed by a high fever, with some difficulty of breathing, which last symptom had been gradually growing worse. I found him propped up in bed in a half-recumbent position, flushed face, brown furred tongue, hot, dry skin, pulse 125, fever heat $104\frac{1}{2}^\circ$, with distressing dyspnœa, with frequent effort to cough. Physical examination discovered about the whole of the posterior of both lungs to be in a state of congestion, as also anteriorly, except the upper lobes. Dr. M. having now arrived, after taking a glance at the patient, we retired in consultation. After hearing his views of the case and his treatment, which consisted of the usual heart sedatives and expectorants, I told him it would take something more powerful, more prompt and decided in its action, to relieve the urgent symptoms in this case, which he did not question. I then proposed venesection, as in my opinion the only remedy that could meet the case, or at least, all other remedies without it would prove useless. He replied, I believe if you bleed this boy he will die, and if you insist upon it, I shall ask to be excused, as I am not willing to share in the responsibility. In this dilemma we agreed tocall in the father and let him decide which of us should take the case; so we did, and Mr. C., an intelligent, strong-minded man, of quick decision, turned to me and said: "Take the case, and do what you think best, for something must be done and that speedily, or my boy can't live." I corded his arm, and opened a bold stream into a wash basin, telling the patient to let me know if he should feel sick or faint; so when he showed symptoms of fainting, I uncorded his arm, lowered his head and shoulders, and the blood stopped. I put him upon tr. verat. viride and tr. gelsemium aa ʒ ij. Dose, 6 to 10 drops every four hours, according as the symptoms might demand, to be given with epsom salts ℥ ss, paregoric and water aa fl ℥ ij. Dose, one teaspoonful, to remove nervous irritation and act on the secretions. With but little addition to, or change in this treatment, there was no more dyspnœa noticeable, a slight tendency to pyrexia the next afternoon, which was easily controlled, and on the third day, when I took Dr. M. down with me to see the patient, we found him sitting in a rocking chair by the fire. Dr. M. asked him if he was not very weak after so heavy a bleeding. It is enough to say that the case made a speedy and thorough recovery.—*Ther. Gazette.*

[To the older members of the Profession, who were formerly accustomed to relieve such cases with the lancet, and who yet occasionally avail themselves of this powerful remedy, the relief in the above case is not new or strange, but an old and familiar fact. While many cases recover without the lancet, there are many, also, that die for the want of it.—*Ed. Rec.*]

**Koch on the Power of Drugs to Arrest the Development of the Cholera Bacillus.**—Koch, from his experiments to ascertain the relative power of drugs to stop the developement of the cholera bacillus, brings out the following facts:

Iodine in solution of 1 to 4,000 has no effect on them, while alcohol in the strength of one part to ten; sulphate of iron, 1-50; alum, 1-100; camphor, 1-300; carbolic acid, 1-400; oil of peppermint, 1-2,000; sulphate of copper, 1-2.500; quinine, 1-5,000; and corrosive sublimate, 1-100,000 were competent to arrest their development. These facts with reference to quinine and corrosive sublimate, are especially noteworthy, as these drugs have proved, clinically, of value in the treatment of cholera. They also show that iodine must prove useless when used on this principle, because of the very small amount capable of being introduced into the system.

Koch finds that the cholera microbe differs also from other pathogenic bacteria by being destroyed by drying, and as the result of further experiments on this point, it was found to be incapable of passing into a permanent state. In this fact he finds further evidence to support his opinion that this micro-organism is not a true bacillus, but is closely allied to the spirillum; and adds that the absence of the permanent state coincides with the experience of the etiology of cholera. The deduction, therefore, is the early annihilation of the disease by preventing its spread through infected water and other material presenting conditions favorable to the life of the germ.—*Maryland Med. Jour.*

**Cocaine.**—Experiments of Frohnmuller appear to show that cocaine is a narcotic, since he was able in fourteen cases to produce sleep by giving large doses (5 grains). What interests us mainly at the present is its properties of a local anæsthetic. In 1868 Moreno showed that reflex movements were abolished for a time when this drug was injected subcutaneously. Von Anrep, in 1880, again demonstrated that the sensibility of the skin was abolished under the same procedure. The same author applied weak solutions to the conjunctiva, and found that it caused a temporary dilation of the pupil, which was increased by adding atropine. It is a strange fact that Anrep did not notice that the conjunctiva became insensible, or if he noticed it he did not recognize the importance of it. The local anæsthetic effect of cocaine seems to have been noticed independently by a number of persons, but it remained for Dr. Carl Koller to discover the local application of cocaine to produce anæsthesia of the cornea and conjunctiva. A preliminary announcement of the observations of K. was made at the congress of German ophthalmologists, which convened at Heidelberg during the month of September, through his friend, Dr. Brethauer. Laryngologists have used cocaine painted over the vocal cords to produce anæsthesia or analgesia of the larynx or pharynx, for the purpose of facilitating examination. This already was a discovery that would make the drug invaluable to specialists, but when Koller discovered its action on the cornea and conjunctiva, it undoubtedly marked an era in opthalmic medicine and surgery that will be as beneficial to suffering humanity as the discovery of chloroform. Until now, reports from clinics, that are to be found in almost every journal, are hasty observations. They only embrace the history of its action in a few cases—experiments that are only a few days old. We sincerely hope, though, that this remedy may not meet with the same result that so many new and apparently efficient drugs meet with.—*Ther. Gaz.*

**Very Small Doses of Calomel in Pneumonia.**—In the Bulletin General de Therapeutique, of July 30, Dr. Droux de Chapois extols the calomel treatment of pneumonia by very minute doses. He prescribes two milligrammes (about one-thirtieth of a grain) every hour for two days. After thus treating over 150 cases, he claims better results than by any of the methods of treatment most vaunted in the text-books. Thus used, the protochloride of mercury has the advantage of not being a weapon that cuts both ways; it produces no violent commotion in the system, but, nevertheless, exercises an incontestable resolute influence over pulmonary hepatization. After twenty-four, or, at most, forty-eight hours, a mild and unctuous moisture ensues over the whole integument; the tongue and mucous membrane of the mouth become moist, the oppression and heat diminish; sometimes a liquid stool after fifteen or twenty doses; finally the fever abates, and bronchial breathing gives place to the crepitant rale redux. It is not claimed that calomel thus given is a specific, but that when in spite of the administration of all the well-known remedies, in place of amendment the symptoms tend to become aggravated, the tongue dry, the skin hot and pungent, calomel given in minute doses every hour, is followed in twenty-four or forty-eight hours, not by profuse sweating, as true sudorifics produce, but by a gentle stimulation of the skin and sebaceous glands, the liver, pancreas, salivary glands, muciparous glands of the alimentary canal and air passages, and the kidneys. After the first day, should the bowels be too loose, the dose is reduced to one milligramme, and if, as sometimes occurs, there should be slight intestinal colics, a little magnesia is given to rid the system of the calomel, when it has become saturated and the desired therapeutic end reached.—*Canadian Practitioner.*

**The Muriate of Cocaine.**—Reports of the use of this salt continue to pour into our eastern metropolitan exchanges, and, almost without exception, they confirm the belief that there has been discovered a drug of immense value as a local anæsthetic, particularly to mucous surfaces. Its action is, however, by no means confined to the eye. It has been used to allay the irratability of the urethra in a case in which the passage of the catheter caused great pain, and under its use one reporter was enabled to perform, with a minimum amount of pain, two operations for the relief of laceration of the cervix uteri, while it is said to have caused such anæsthesia of the skin over a boil as to have permitted its free incision without suffering.

Naturally, the wonderful results above hinted at have caused an immense demand for the drug, and, we are informed, the stock on hand in our American drug houses was promptly exhausted. Supplies are, however, now on the way from Europe (Merck's laboratory), and in a few days at furthest there will, doubtless, be enough in stock to meet immediate demands. The price of the salt is necessarily high, the coca erythroxylon yielding only a fiftieth of one per cent. of the alkaloid. The price will, therefore, probably not get much below fifty cents a grain. A two-per-cent. solution would, at this price, cost about $5.00. This price will interfere with its general use.—*Medical Age.*

**The Grit Cure.**—According to an exchange, a *restauranteur* who had contracted dyspepsia by his gluttony conceived the idea that chickens and other fowls escaped dyspepsia notwithstanding their miscellaneous diet, by means of the large quantities of sand, gravel, plaster, etc., which they consume. He accordingly resolved to imitate them, and, as the reporter goes on to say, "used marble-dust instead of salt on his beefstake, and filled his pepper-box with sea sand. Receiving so much benefit from these kinds of grit, he proceeded to swallow gravel and pieces of plastering. In a few months he was entirely cured. He can now eat as much as an ostrich, and never suffers on account of the kind or amount of food he consumes. He is thankful that he went to the chicken and considered her ways, and recommends the gravel remedy to all who are suffering from indigestion."

The remedy certainly posseses the merit of cheapness, as sand is abundant everywhere; and compared with the avegage dyspepsia-curing nostrums advertised in the newspapers and on every fence and large rock by the wayside, it may be regarded as harmless, although we should be loth to recommend the average dyspeptic's stomach to undertake a diet of small rocks and brick-bats.—*Good Health.*

**New and Successful Treatment for Tapeworm.**—Under the above title, Dr. Howard Pinkney, writing from Sharon Springs to the Med. Record, describes his experience with the oil of the pine needle, made from the *pinus punilio.* A hall-boy of the hotel had suffered for five years from tapeworm. He had been treated for four years in New York, but had never succeeded in getting rid of over four feet of links at a time. Dr. Pinkney not being able to get any male fern, pelletierine, or pumpkin seeds, therefore tried the following experiment:

"The patient fasted from breakfast, and at 9 p. m. he was given one teaspoonful of the oil of the pine needle in half a glass of milk. The following morning, as there was no perceptible action of the medicine, the dose was doubled. This, the boy said, had a most agreeable taste. One hour later he took a dose of castor oil, and in the course of two hours after this he passed an entire tænia solium measuring fifteen feet six inches in length, and one-half inch at its broadest part, gradually tapering down to almost a thread. To be positive that none remained behind, he was given two teaspoonfuls more. but no sign of any worm or part thereof passed. "This oil." writes Dr. Pinkney, "contains no turpentine, is fragrant in its odor, and when mixed with milk very agreeable to the taste. It produces no strangury, tenesmus, or other unpleasant or distressing symptoms. The patient can generally pursue his ordinary avocation." The correspondent would be pleased to know if any of our readers have ever read or known of its use in similar cases.—*Med. and Surg. Rep.*

**Witch Hazel in Hæmorrhage.**—Will D. Christy, M. D., Afton, Iowa, writes: "I notice two short articles in your last number on the use of hamamelis, or witch hazel, in the treatment of varicose veins and menorrhagia, and am surprised to see this old and truly good remedy spoken of as something new. I know of no other remedy that has the property of being comparatively harmeless, and yet so potent in relieving so many different derangements of the vascular system. It is the only true venous tonic in our materia medica, and has the power of controlling hæmorrhage of a venous character beyond any remedy I have ever tried. Last fall I had three cases of typho-malarial fever that had hæmorrhage from the bowels, and the witch hazel was the only remedy used for that trouble. I gave the distilled extract in teaspoonful doses every hour for two or three doses, and then three times a day until all danger of a recurrence was past. In no case was there any loss of blood after the second dose. I have used it frequently for the hæmorrhage following abortion, and, in fact, put more faith in it than in ergot or lead and opium in such cases. As a local application to contusions it has no superior. I have tried the fluid extract several times, only to be disaspointed, and now rely solely on the distilled extract."—*Therap. Gaz.*

**Titanium or Cimicifuga.**—In the October number of the Medical Brief I noticed an article by Dr. M. M. Griffith, bringing the carbonate of titanium before the profession as a positive emmenagogue in amenorrhœa. The article above mentioned, it appears, has created quite a boom in the titanium market. In a little time the market will probably be overstocked, as titanic acid will be obtained from rutile, which is found in the limestone regions; and from titanic iron, found in the iron regions of Pennsylvania.

His attention was called to its medical properties by reading a French medical journal several years ago, in which it was recommended as a stimulant to the sexual organs, with the expectation of eventually producing barrenness.

He claims to have obtained very good results from its use in combination with aloes; and succeeding in relieving amenorrhœa with certainty. The stimulating effect may be, and often is desirable; but is the second property, or barrenness, to be encouraged among our American women?

I read his article a second time, but did not, however, feel as though titanium was required if I could obtain cimicifuga. For over twenty years I have used cimicifuga racemosa, or black snake root, and produced almost as much as he claims for titanium, with the exception of produeing barrenness, as it has the opposite effect, of increasing the tendency to fecundity. In cases of retention, I frequently give the following: Saturated Tinct. Cimicifuga Rac ʒ j, water ℥ iv, a teaspoonful every two or three hours, commencing a week before the menstrual epoch. If the case is one likely to be tardy and require increased doses, I usually on the day before, or the day the discharge is due, increase to a drachm of the tincture thiee times daily. A short time ago a girl about fifteen years of age came from the seaside to be treated for amenorrhœa, with tuberculosis of both lungs developing rapidly. She had been treated by several skilled brethren for upwards of two years, at her home, and they retired from the conflict unsuccessful.

In this case I used the macrotin or cimicifugin in water; and in two weeks her long looked for visitor arrived. At the next period she overrun her time five or six days without having taken any cimicifuga, when a call was made, the condition of the patient stated, and a teaspoonful of the tincture given in the evening. The following morning there was sufflcient evidence that the medicine had acted as desired.

A few years ago I gave the cimicifuga to a lady patient who had pnssed the change for over a year, without any evidence of a return of the catamenia, and in two or three days it was re-established. It was unintentional, as the medicine had been given for other purposes. I have had the same results in more than one case, similar to the last one, and in many cases similar to the first mentioned. When there is suppression in married ladies from natural causes it can not, and should not, be depended upon, although I can recall more than one who used the ordinary tincture in dessert or tablespoonful doses, and could procure a good free discharge at any time. If given in doses of one or two drops every three or four hours for a week or two previous to the menstrual period, it will prevent those heavy, dragging pains incident to congestion of the pelvic viscera, and produce a normal condition of the organs. I always discontinue it a day or two previous to the epoch, if menorrhagia is anticipated, as the tendency is to augment its flow. I prefer the saturated tincture made by maceration, using eight ounces of the green or very recently dried root to a pint of alcohol of 76 per cent., but frequently use the macrotin or cimicifugin of the first decimal trituration, and get very nearly the same results, but not quite so promptly, giving from two to five grains of the triturate.—*G. W. M. Calver, New Jersey, in Ec. Med. Jour.*

**Does Hereditary Syphilis Exist?**—In a report of a clinical lecture on syphilitic sequelæ by Dr. Fessenden N. Otis (*Phila. Med. Times*) the following views of the lecturer are worthy of being transcribed: Mr. Hutchinson. generally conceded to be the greatest English authority on syphilis, distinctly supports the germ theory of syphilis, and carries it to the legitimate conclusion that the disease is confined in every instance to the individual organism infected, and hence that it is incapable of being acquired or communicated through hereditary transmission: in other words, that there is no such disease as hereditary syphilis, any more than there is an hereditary small-pox, and that in every case of syphilis the disease is acquired through contact with a disease-germ of syphilis in an organism previously free from that disease, whether it occurs in the ovum, the embryo, the fœtus, the infant, or in the adult. * * * The mother must first acquire the disease; and it is only through the disease-germs of syphilis circulating in her organism that the product of conception can be infected before birth. * * * It is undoubtedly the fact that much disease in fœtal and in infantile life results from pre-existing disease, the legitimate sequel of syphilis in the organism of the mother; but that any syphilitic disease proved to be such by its power to transmit syphilis has been communicated to healthy persons by infants conceived after the active or contagious stage of syphilis in the parents has passed, there is no well authenticated evidence to prove. And this stage * * * has been shown, by ample testimony, not to extend over a period of three or four years.—*St. Louis Med. and Surg. Jour.*

**Malarial Fever.**—Dr. Roberts Bartholow, in a running comment on cases in his clinic reported in the College and Clinical Record, says; "This little girl has suffered from periodical attacks of fever, accompanied with vomiting and headache, and the case was considered one of ordinary sick-headache or megrim. It was, however, clearly a case of malarial toxæmia, and we had to deal with an attack of intermittent fever.

In order to prevent these periodical seizures, we gave quinine in massive doses, but something more was required. It was necessary to pay attention to the condition of various organs, especially of the liver and spleen, for, as I pointed out, if the functional disorders of these organs is not corrected, you cannot prevent the recurrence of the attacks, no matter how much quinine may be given. If there be a bacillus malariæ responsible for these attacks, this is an explanation of their recurrence, for if the liver and spleen be affected, as we know they are in cases of chronic malarial toxæmia, obviously these organs are in a condition to favor the development of the parasite, or these organs may be in a pathological state from the presence of the parasite. Whether this theory be true or not, or whether there be a bacillus malariæ or not, it is a fact that in cases of chronic malarial toxæmia the attacks cannot be broken up unless the condition of the liver and spleen be restored to the normal. I attempted to remedy this trouble by the following combination of remedies:

℞ Extracti ergotæ .................................. gr. j
Ammonii iodidi .................................. gr. j
Ferri arseniatis .................................. gr. 1-20

M. Ft. pil. No. j, which is to be given three times a day.

The attacks have not occurred since she was put upon this treatment, and the child's condition is much improved. It will not be necessary to administer any longer the massive doses of quinine, but we shall continue the pill for at least two weeks.—*St. Louis Medical and Surgical Journal.*

**"Digestive Chologogue."**—

℞ Sulphate manganese, } aa .......................... ʒ ij

Mur. ammonia, }

Fl. ex. ipecac .................................. gtt. xx

Glycerin .................................. ℥ j

Aqua dist. q. s .................................. fl. ℥ iv.

M. Ft. sol. S. Teaspoonful three times a day, just after meals.

For chronic hepatic derangements, malarial dyspepsia, etc. An appetizing tonic for consumptives and chronic catarrhal inflammatory diseases of the respiratory mucus membranes.

This is very efficacious for chronic hepatic troubles. Here in the long hot summers our livers are prone to get diseased, and too often fatally so; hence we must give much attention to hepatic therapeutics. The "digestive chologogue" is well suited for continuous use for weeks or months at a time, and when thus used will often relieve hemorrhoids more or less permanently, and if other hemorrhoidal drugs be conjoined, many bad cases of hemorrhoids may be promptly relieved, and within two to four months cured, and in the meantime the general health well established and the patient made strong and hearty. The "digestive chologogue" is well suited to consumptives and those whose respiratory mucus membranes are affected. In these classes of patients the hepatic and other glandular functions are generally quite sluggish; and said remedy will increase the appetite and increase the heat-making power of those who are chilled with every cold breeze.—*Q. C. Smith, in New Eng. Med. Month.*

**The Treatment of Sprain by the Elastic Bandage.**—This method of treating sprains has recently been recommended by Mark See. It is the only method which fulfills the two indications:

1. To cause as rapid absorption as possible of the blood extravasated around the joint (a lesion which controls all the other symptoms, such as pain, swelling, difficulty of movement, etc.)

2. To favor cicatrization of the torn ligaments and ruptured parts by complete immobilization.

The antiphlogistics and blood-letting, formerly advised by Hunter and Guersant, only partially fulfill the former indication. There is the same objection to the movements which Ribe and Bonnet advise for the injured joint. The refrigerants and cold water baths advised by Baudens cause contraction of the tissues around the joint and dispel the inflammation, but they are not favorable to the absorption of the infiltrated fluids. Even massage, though superior to the other remedies just mentioned, fulfills only the second indication; furthermore, it is inconvenient and requires much patience and time, and between the seances of manipulation the swelling reappears and the pain returns. It is true that massage has the advantage of removing the extravasated materials from the region of the joint toward the more vascular portions of the limb, where they are more easily absorbed. But the elassic bandage has this advantage in a greater degree, since its action is continuous. Finally, and above all, it favors immobilization of the joint, which is impossible during massage, and without which it is almost impossible to get cicatrization of the torn structures and complete recovery in sprains of any intensity. The bandage should be applied to the skin itself, care being taken to fill up the flat and depressed places with wadding, so as to give a uniform surface around the joint for the bandage to act upon.—*Revue de Therapeutics*, *July 15*, *1884*.

**Asthma.**—Dr. Griswold (in Louisville Med. News) reports cdses of asthma relieved by chloral Of his first case he writes: "The outlook ahead had no hope in it. Sitting down to cogitation over this case, with a knowledge of a large share of the drugs heretofore prescribed for its relief with entire failure, the conclusion was legitimate that there was little or no use going over again the same treatment; something new had to be thought of. More as a result of reasoning upon the case, and upon the vexed question of the pathology of asthma, than as a mere empirical happy thought, the hydrate of chloral presented itself as worth trial. Soliciting experiment, permission was accorded, and I gave the woman a four-onnce bottle of a solution of chloral, fifteen grains to the dose once in four hours till relief was obtained, and then three times in twenty-four hours till it was all taken. The effect at the first dose was very marked; inside of twenty-four hours there was entire subsidence of the distress, and after the quantity prescribed was taken as directed the patient expressed herself as feeling cured. What may seem still more remarkable is the fact that from thence on the woman has been so free from the attacks of her old enemy that she has not had to use any drugs to repel him.

**Puerperal Septicæmia.**—Thomas, in Med. News, remarks: "The germ theory has done more for obstetric medicine than what I have here alluded to. It has revolutionized the treatment of that variety of septicæmia which has been called puerperal fever. No longer do we depend, in the treatment of this affection, upon quinine, opium and the application of emollients over the abdomen By intra-uterine injections the cavity of the uterus is thoroughly and repeatedly washed out with solutions of the bichloride of mercury 1 to 2000 or with a two and a half per cent. solution of carbolic acid. Surely no one who has experience in the new and the old methods will cavil at my statement that a great improvement has been effected by the former.

Were I called upon to sum up the treatment of a declared undoubted case of puerperal seyticæmia, marked by the usual symptoms of pulse of 120, temperature 105° or 106°, which would meet the requirements of our time, I should give it categorically thus:

1. Quiet all pain by morphine hypodermically.
2. Wash out the uterine cavity with antiseptics.
3. Lower the temperature at once below a hundred, not by the barbarous method of the cold bath, but by the far better one of the coil of running water.
4. Feeding the patient upon milk and nothing else, unless some good reason exists for changing it.
5. Exclude from her room all except the nurse and the doctor, keeping her as quiet as possible.

**Coca in Atrophy of the Retina.**—Dr. Marshall, in Louis. Med. News., says: William Tracie, white, aged forty, was for two months under treatment in the eye and ear ward of the Louisville City Hospital for so-called atrophy of the retina. His sight had uniformly improved under the use of strychnia hypodermically. Beginning with one-forty-eighth of a grain, the dose was progressively increased until a twelfth of a grain was given morning and night. Upon the plea af business he was allowed to leave the hospital, with the promise of returning in a few hours. He stayed away for several days, and was finally returned to the hospital with a well-marked delirium tremens, his impairment of vision having increased under the dissipation.

According to my custom in dealing with such cases, he was put on dram doses of the fluid extract of coca, administered every two hours. At the end of twenty-four hours he called attention to a marked improvement in his sight. The drug has been continued, and his sight is improving much faster under its use than it did while he was taking the strychnia.

From the foregoing it would seem that coca is likely to do good service as a substitute for strychnia in the eye diseases arising from the abuse of whiskey and tobacco.

It is my hope that the profession may be encouraged by this favorable report to give the drug further trial in eye troubles characterized by impairment of vision, and especially in that affection which is called by the Germans atrophy of the retina.

**Tape Worm.**— Dr. Bernard (in Med. Times) reports twenty cases of tape worm relieved as follows: "One drop of croton oil and a drachm of chloroform are suspended in an ounce of glycerine, and administered in the morning before breakfast. The only preparatory treatment consists of a half ounce of Rochelle salt given the preceding evening, which, although not necessary for a cure, facilitates the examination of the evacuations, prevents the breaking of the worm by hard fæces and allows it to pass more quickly through the intestines after becoming detached. The chloroform produces no bad effects; the slight giddiness and drowsiness sometimes noticed was relieved by the recumbent posture and disappeared when the croton oil commenced to operate. The oil acts rapidly, the bowels being moved in about an hour after its administration, and any tendency to diarrhœa or intestinal irritation is readily checked by bismuth and opium after the worm has been expelled. In one case the chloroform alone was efficient in bringing about the expulsion of the worm; but the fact that the worm is always expelled alive, showing that the chloroform, while compelling it to relinquish its hold, is not sufficient to kill it, renders the administration with it of a drastic purgative of rapid action advisable. The author concludes by stating that in the cases treated successfully in this way, other remedies had been unsuccessfully employed. The patients agreed that the remedy was readily taken, that its immediate effects were by no means unpleasant, and that the treatment did not leave them prostrated.—*Maryland Med. Jour.*

**Silicate of Sodium Bandage.**—Dr. E. O. Bardwell speaks in the highest terms of commendation of the silicate of sodium paint to bandage for a fixed dressing in fractures of the long bones.

It is of a syrupy consistency, and is readily applied by painting with a common flat varnish brush after applying a roller bandage, protecting the joints and bony prominences with cotton. Other bandages are then applied and painted in the same manner until four or five thicknesses are in position. If necessary, strips of roller may be laid lengthwise between the bandages and painted with the solution. The bandage so applied is more uniform in thickness and more cleanly than those of starch, dextrine or plaster of Paris, and does not contract or dry like those.—*St. Louis Cour. Med.*

**A Mode of Curing Cancer.**—Dr. H. M. Lawson, of Cuthbert, Ga., who is described by the editor of the Southern Medical Record as "a modest though worthv practitioner," announces a cure for cancer. It consists, as we understand, in the heroic use of srsenie combined later with caustic applications. In one patient suffering from epithelioma on the left temple, one-fourth of a grain of arsenious acid was given, and gradually increased to three-fourths of a grain.—*N. Y. Med. Jour.*

**Notes on the Use of Cocaine.**—The Baltimore Surgeons have been using cocaine extensively in eye and venereal diseases. The reports are as enthusiastic as any published in this city. Dr. Michael found it very useful in opening buboes and cauterizing venereal sores. Dr. E. E. Holt, of Portland, Maine, reports sucsessful application of cocaine in eye cases (Boston Medical and Surgical Journal). Dr. W. E. Ground, of Toledo, Ohio, has also had uniform success with the drug. In Vienna the drug is being more and more used. Recent reports upon it have been made by v. Schotter, Storck, Konigstnin and Jelinck. The solutions employed are stronger (ten to twenty per cent.) than those which have been found adequate here. England and France have, as yet, had little to say about it, but wherever used the reports are unanimous in its favor.—*N. Y. Med. Rec.*

**Cholera.**—Dr. Robert Sproule, a member of the Cholera Commission in India, says (in Medical World):

Opium, in the earlier stages, combined with sulphuric acid and good brandy or sulpuric ether, is, in my experience, the most reliable medication; and even when there is no diarrhœa, the following will be found highly efficacious:

℞. Opii. Tinct.......................................ʒ iss

Ac Sulph. dil.......................................ʒ iv

Spt. Vini. gall.......................................ʒ ij

Aq. Camph. vel menth pip ad.......................ʒ iv

M.—One table spoonful every half hour till relief is afforded. The dose of opium may be varied, and more diffusible stimulants given, at discretion.

In the seccnd stage, or that of prostration and collapse, I found that a hypodermic containing morphia ⅛ grain, and atropia 1-60 grain, often acted like magic in restoring the vital powers, and in many cases the patient in a few moments awakened, as it were, and shortly afterwards, I will say in ten or fifteen minutes, and sometimes even in five minutes, was able to take stimulants and medicine per orem. I have great faith in the use of morphia and atropia hypodermically in this stage of cholera. Quinine and calomel were nearly always given once or twice after the hypodermic injection, and always with happy results, and, if the diarrhœa continued, it was controlled by lead and opium *in solution*, or by opium and sulphuric acid.

The drink to assuage the thirst, which was used and found to agree best, was iced beef-tea or iced milk and water, and either of these was given ad libitum. I gave the beef tea sometimes with a little wine, but this was not liked, though I had reason to think it did more good than the beef-tea alone.

Rice, arrow-root, milk, sago, etc., with plenty of good soups and a liberal allowance of wine, were the means employed to hasten convalesence.

The patient was always confined to the recumbent position though not necessarily in bed, for a few days after a moderate attack.

**Hops and Cider for the Relief of Exophthalmic Goitre.**—Before the Sheffield Medico-Chirurgical Society, November 20, 1884 (Medical Press), Dr. Baldwin exhibited the man shown by him a year ago with this affection. He was then unable to lie down, and apparently was in a most critical condition. During the past summer he has been roughing it, hop gathering, etc., drank a quantity of cider, and is now much better, and able to lie down in moderate comfort.—*Louis. Med. News.*

**Reduction of Dislocation of Humerus.**—The Southern Clinic thus gives Dr. Gissler's method of reduction.

"In my cases the patients do not even have to sit down and I operate thus:

1. The elbow is pressed against the abdomen and then gently drawn outward until resistance is met with.

2. The forearm is then raised as high as possible toward the opposite shoulder.

3. Then the whole arm is drawn outward and the operation is finished."

This is a valuable addition to our knowledge of the operations which are daily needed. It is simple, accurate and may be of use."

**Another "Specific"**—And now comes the ex-house surgeon of Bellevue Hospital, Dr. Seelye, of Massachusetts (New York Medical Journal), and says oil of wintergreen (gaultheria) is a specific for acute rhematism, the more severe the symptoms, the more joints affected, the higher the temperature, the more prompt and satlsfactory the action of the remedy. And extensive experimentation of eighteen month's duration on three wards of the Bellevue, demonstrated the vast superiority of this over the salicylate and all other treatment. Dose, 10 minims in a half drachm eaeh of glycerine and water, every 2, 3, 4 and 6 hours, according to the severity and nature of the case.—*Curier-Record.*

**Chronological History of the Discovery of Disease Germs.**—Dr. Andrew Smart, of Edinburgh, gives the following (Brit. Med. Jour.) as the chronological order of the discovery of disease germs:

1. Rinderpest-germ, Dr. Smart, September, 1854; 2. Relapsing fever germ, Obermeier, 1868; 3. Anthrax germ, Koch, about 1864; 4. Vaccine germs (probably analogous to small-pox germs not yet discovered), Sanderson & Chanveau, 1869; 5. Filaria Sanguinis Horminis, Manson, 1881; 6. Typhoid fever germ, Ebert, 1880; 7. Bacillus tuberculosis, Koch, recently; and 8. Cholera germ, Koch, recently.—*Maryland Med. Jour.*

**Terpine.**—Under this name M. Lepine gave an account at the Lyons Societe des Sciences Medicales (Lyon Medical, November 16th), of a new therapeutical agent produced by a chemical combination of turpentine, alcohol and nitric acid. In doses of from 29 to 40 centigrammes he has found it very useful in chronic, and even in sub-acute, bronchitis, greatly facilitating expectoration. Advantage has also been derived from it, in the same or similar doses, in some cases of chronic nephritis. It is a diuretic acting directly on the renal epithelium, requiring to be used with circumspection.—*London Med. Times.*

**Hay Fever—Valer. Zinc and Assafœt.**—Dr. Morell Mackenzie considers pills containing one grain of valerianate of zinc and two grains of assafœtida each very valuable in hay fever.—*Med. Chron.*

## Figures and Tables

**Figure f1:**